# Supplementary material for: Causal Relationship between Aging and Anorexia Nervosa: A White-Matter-Microstructure-Mediated Mendelian Randomization Analysis
Source: Biomedicines. 2024 Aug 16;12(8):1874. doi: 10.3390/biomedicines12081874 (PMC11351342; doi:10.3390/biomedicines12081874)
Supplement: Supplementary file 1 [file biomedicines-12-01874-s001.zip › Supplementary Figure/Figure S4/Singe SNP plot of MR analysis for TL to ubm-b-1840.pdf]

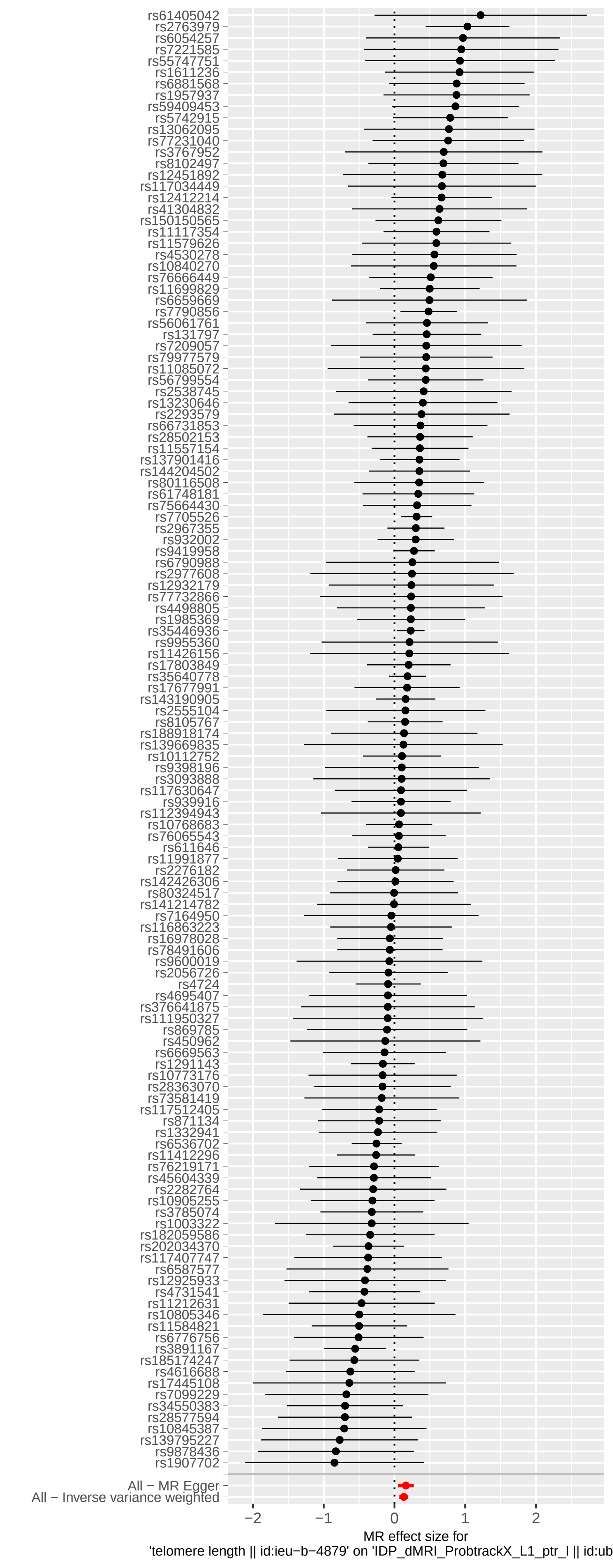
rs61405042
rs2763979
rs6054257
rs7221585
rs55747751
rs1611236
rs6881568
rs1957937
rs59409453
rs5742915
rs13062095
rs77231040
rs3767952
rs8102497
rs12451892
rs117034449
rs12412214
rs41304832
rs150150565
rs11117354
rs11579626
rs4530278
rs10840270
rs76666449
rs11699829
rs6659669
rs7790856
rs56061761
rs131797
rs7209057
rs79977579
rs11085072
rs56799554
rs2538745
rs13230646
rs2293579
rs66731853
rs28502153
rs11557154
rs137901416
rs144204502
rs80116508
rs61748181
rs75664430
rs7705526
rs2967355
rs932002
rs9419958
rs6790988
rs2977608
rs12932179
rs77732866
rs4498805
rs1985369
rs35446936
rs9955360
rs11426156
rs17803849
rs35640778
rs17677991
rs143190905
rs2555104
rs8105767
rs188918174
rs139669835
rs10112752
rs9398196
rs3093888
rs117630647
rs939916
rs112394943
rs10768683
rs76065543
rs611646
rs11991877
rs2276182
rs142426306
rs80324517
rs141214782
rs7164950
rs116863223
rs16978028
rs78491606
rs9600019
rs2056726
rs4724
rs4695407
rs376641875
rs111950327
rs869785
rs450962
rs6669563
rs1291143
rs10773176
rs28363070
rs73581419
rs117512405
rs871134
rs1332941
rs6536702
rs11412296
rs76219171
rs45604339
rs2282764
rs10905255
rs3785074
rs1003322
rs182059586
rs202034370
rs117407747
rs6587577
rs12925933
rs4731541
rs11212631
rs10805346
rs11584821
rs6776756
rs3891167
rs185174247
rs4616688
rs17445108
rs7099229
rs34550383
rs28577594
rs10845387
rs139795227
rs9878436
rs1907702
All − MR Egger
All − Inverse variance weighted
−2
−1
0
1
2
MR effect size for
'telomere length || id:ieu−b−4879' on 'IDP_dMRI_ProbtrackX_L1_ptr_l || id:ub
